# COVID-19 vaccination hesitancy among Iraqi general population between beliefs and barriers: An observational study

**DOI:** 10.12688/f1000research.110545.1

**Published:** 2022-03-18

**Authors:** Laith G. Shareef, Ali Fawzi Al-Hussainy, Sajid Majeed Hameed

**Affiliations:** 1 Department Of Pharmacy, AL-Esraa University College, Baghdad, 10011, Iraq; 2 Clinical Pharmacy Department, Baghdad Medical City, Baghdad, 10011, Iraq; 3 Clinical Pharmacy Department, Medical Rehabilitation Hospital, Tikrit, 34001, Iraq

**Keywords:** Perception, attitudes, Iraq, COVID-19, coronavirus, vaccine acceptance, SARS‑CoV‑2, vaccine hesitancy

## Abstract

**Background:** Vaccine apprehension poses a serious threat to global health. While there has been a tremendous global effort to create a vaccine against coronavirus disease 2019 (COVID-19), little is known about its reception in  Iraq. Therefore, we sought to examine COVID-19 vaccine acceptance, hesitation, and related elements in the Iraqi population.

**Methods:** Between the 19th of May and the 22nd of September 2021, a descriptive, cross-sectional web-based survey was conducted employing a quantitative approach. Respondents from both sexes aged 18 years and above who live in Iraq and agreed to participate were included. An anonymized online structured questionnaire was designed based on data from prior research on vaccine  hesitation in general,  and COVID-19 vaccination reluctance specifically.

**Results:** A total of 1221 eligible participants from various regions in Iraq actively participated in the short web-based questionnaire. The overall acceptance rate of the COVID-19 vaccine was 56.2%, with a greater acceptance rate among younger male participants (p<0.05). Marital status had no significant association (p=0.834). Urbanization influenced the acceptance rate significantly (p=0.002). The barriers to receiving the COVID-19 vaccine were exemplified by the vaccine not being evaluated for a sufficient period in 51.4% of the responses, as well as concerns about future side effects in 76.6% of the responses and a lack of efficacy in 55.7% of the responses. The Pfizer-BioNTech vaccine received 39.6% preference and participants confidence, followed by the Oxford/AstraZeneca vaccine at 18.1% and the Sinopharm vaccine at 14.6%.

**Conclusions:** COVID-19 vaccination apprehension was discovered in almost half of the study population. Lack of understanding about vaccination eligibility, anxiety about adverse events and vaccine efficacy, and distrust in the government were independently predictive of vaccine hesitation.

## Introduction

The coronavirus disease 2019 (COVID-19) pandemic caused by the emerging severe acute respiratory syndrome coronavirus 2 (SARS-CoV-2) virus, is still causing turmoil around the world. This pandemic seems to have had a negative influence on living standards and placed a strain on health care organizations. ^1^ Diverse chemotherapeutic and biological treatments have already been tested in COVID-19 cases, but none have been shown to provide a definitive therapeutic efficacy. ^2^ With little effectiveness, many preventive public health interventions such as quarantining, practicing hand hygiene and cough etiquette, as well as physical separation are all measures that were instated. ^3^

When vaccines were introduced during a previous influenza pandemic, the acceptancy level ranged between 8% and 67% as stated in a systematic review by Larson *et al*., in 2018. ^4^ According to a meta-analysis by Xiao and Wong, the acceptance rate in the United States in 2020 was 64%. ^5^ Also, in the United Kingdom, about half of survey respondents said they were willing to try the H1N1 influenza vaccine according to a report by Chan *et al*., in 2015. ^6^ During the 2014 Avian influenza A(H7N9) outbreak in China, 50.5%of research participants were vaccinated with the A/H7N9 vaccine as soon as it was available. ^7^ In Beijing, 59.5% of research respondents aware of H7N9 expressed that they would be willing to receive a future influenza A (H7N9) vaccination. ^8^ Vaccine acceptability and need are multifaceted and context-dependent, altering over time periods, geography, and the beliefs influencing the scope of the community. ^9^ According to a survey performed in Ireland, medical personnel ignored seasonal flu immunization mainly because of misapprehension regarding the efficiency and dependability of the vaccine. ^10^ In China, demographic characteristics and public perceptions are major factors of vaccine uptake. The primary determinants of vaccine acceptance in Hong Kong are anxiety levels and vaccine experience. ^6^ In the United States, the most important determinants of influenza vaccination adoption were anticipated vaccine efficiency, cultural influence, and insurance coverage. ^7^ Higher introversion was related to poorer vaccination uptake in studies conducted in the United States, while greater confidence was linked to greater vaccination uptake. ^11^ A study on parental opinions of pediatric age group immunizations in the UAE discovered that just 12% of respondents were against pediatric immunizations, according to the report, vaccination safety (17%), adverse reactions (35%), and too frequent injections (28%) were all critical factors in vaccine reluctance. ^12^ Participant willingness to get the COVID-19 vaccine was higher among those who had previously received a seasonal flu vaccine. ^13^

Thus far, several vaccines have been created, with some licensed and others continuing in clinical studies. Notably, the Pfizer-BioNTech, Moderna, and the Oxford/AstraZeneca vaccinations have all been licensed for urgent application and are now in use in a number of countries, including Iraq. ^14^^,^^15^ Despite significant progress in vaccine research, public acceptability of the COVID-19 vaccine remains a significant barrier. ^16^ As per the World Health Organization (WHO), vaccination reluctance is among the 10 leading threats to global health, but it is being aggravated mostly by rising conspiracy theories surrounding COVID-19 and vaccines. ^17^ As a result, we sought to examine the acceptance, hesitation and related elements around the COVID-19 vaccine in the Iraqi population.

## Methods

### Ethical consideration

This study was approved by the Al-Esraa University College Ethics Committee (approval no. 506). The Al-Esraa University College Ethics Committee waived the need for consent for the acquisition, interpretation, and publication of the prospectively collected anonymous data for this non-interventional research. Before participating in the survey, individuals were allowed to decline participation in the questionnaire. Participation in this study was entirely voluntary, free of coercion, and uncompensated. Participants provided informed, written consent as part of the questionnaire. Throughout the study, anonymity and confidentiality were maintained. All data were saved in a secure file, with only the researchers in charge of the questionnaire accessing the information. The study was carried out according to the World Medical Association’s Declaration of Helsinki. ^18^

### Study design

This was a descriptive, cross-sectional web-based survey performed between the 19th of May and the 22nd of September 2021, using a quantitative approach.

### Eligibility criteria

Respondents from both sexes aged 18 years and above who live in Iraq and agreed to participate were included. Simple random sampling was the method of selecting participants in which the author chose a subset of participants randomly from a population. Each individual in the population had an equal probability of being considered.

### Bias

The survey was disseminated through a variety of electronic means, including social media and e-mail. Therefore, any concerns regarding sample bias and generalizability may be ameliorated since the form could have been filled out by anybody with an internet connection. Furthermore, the author asserts that there are no instances of reporting bias or performance bias in their findings.

### Study sample size

The sample size calculation was based on prior reports of COVID-19 vaccination hesitancy or refusal, ranging from 6% in Egypt to 13.9% in Italy, 23% in the United States, 30.5% in Malta, and 51.4% in Iraqi Kurdistan. ^19^^,^^20^ This resulted in a sample size of 962 people, equating to the lowest prevalence, relative precision of 25%, and alpha error of 5%. The sample size was determined using the equation N = Zα2P (1 − P)/d2, where = 0.05 and Z = 1.96, and the permissible margin of error for proportion d is calculated to be 0.1. When we received 1221 completed surveys, we decided to end the survey.

### Questionnaire description

Using data from prior research on vaccination reluctance in general, and specifically the COVID-19 vaccine, ^21^^–^^26^ an anonymous online structured questionnaire was developed. ^43^ The set of questions was divided into three sections: the first contained basic demographic information as well as an evaluation of understanding and source of knowledge concerning COVID-19 vaccine, the second contained an assessment of attitudes toward the vaccine, and the third contained details regarding vaccination barriers. This questionnaire was made available online using Google Forms.

### Study variables

The sociodemographic variables of sex, age, marital status, educational level, permanent resident, and sources of knowledge about coronavirus and COVID-19 vaccinations were included as independent key predictors, while the acceptance, hesitation, trust, and perceptions concerning COVID-19 vaccination were dependent variables.

### Data analysis

For cleaning and coding, submitted questionnaire responses were exported from Google Forms to Microsoft Excel 2019 (Microsoft Excel, RRID:SCR_016137). The processed data were transferred to IBM SPSS software for Windows, version 25 (IBM SPSS Statistics, RRID:SCR_016479) for analysis. Means (standard deviations) and percentages were used to summarize numerical data. For parametric and non-parametric data, the median (interquartile range) was used. Categorical data were collected. Frequency and proportions were used to summarize the data. Relationships between independent factors and dependent variables were determined using chi-square test or Fisher’s exact test, and logistic regression analysis was used to evaluate variables. p<0.05 was considered statistically significant. When dealing with missing data, we relied on two primary methods for eliminating data: list-wise and deleting variables. Listwise method: This strategy deletes all data for an observation that includes one or more missing values. Only observations with a full set of data are subjected to the analysis. Dropping variables: If data are missing for over 60% of the samples, it may be best to remove if the variable is insignificant.

## Results

A total of 1221 eligible participants (aged 18 and over) from various country regions actively participated in the short web-based questionnaire. The study population was mainly concentrated in urban areas (71.2% live in urban areas). Furthermore, a larger portion of responders within the age range of 18–29 years represented 40% of the study participants, and the ratio of male to female participants was 0.94:1, where the female percentage was 51.5%. A total of 719 (58.9%) were married. Most study participants held an academic degree (677, 55.4%). The overall acceptance rate was 56.2%. This research showed a higher acceptance rate of COVID-19 vaccination in younger, male participants (p<0.05). No significant association was demonstrated regarding marital status (p=0.834). Geographic area significantly impacted the acceptance rate where most of the respondents lived in urban areas (p=0.002). The level of education seemed to have no noticeable effect on vaccination acceptance rate (p=0.238) ( Table 1). ^42^

**Table 1.** Sociodemographic backgrounds of the study population and its association with the COVID-19 vaccine acceptance rate.

Sociodemographic characteristics		n (%)	COVID-19 vaccine acceptance		p-value
			Yes	No	
Age	18-29	488 (40.0)	254	234	0.041
	30-49	454 (37.2)	278	176	
	50-64	201 (16.5)	112	89	
	>65	78 (6.4)	42	36	
Sex	Male	592 (48.5)	349	243	0.033
	Female	629 (51.5)	337	292	
Marital status	Single	395 (32.4)	226	169	0.834
	Married	719 (58.9)	402	317	
	Separated or widowed	107 (8.8)	58	49	
Geographic areas	Urban	869 (71.2)	513	356	0.002
	Rural	352 (28.8)	173	179	
Education	Primary school graduate	167 (13.7)	93	74	0.238
	Secondary school graduate	173 (14.2)	87	86	
	Diploma or bachelor’s degree	677 (55.4)	382	295	
	Higher education (MSc or PhD)	204 (16.7)	124	80	

COVID-19, coronavirus disease 2019.

Most of the participants did not have an underlying medical condition (798, 65%), 574 (47.0%) participants had been positive for COVID-19 in the past, while 815 (66.7%) reported the occurrence of COVID-19 infection in family members, from the total number of respondents 880 (72.1%) had children. The association of the above parameters with the vaccination acceptance rate showed that healthy people without a chronic illness had a higher acceptance rate of vaccination (p=0.002), patients with a past medical history of COVID-19 infection were accepting the vaccine at a significantly higher rate than patients without a history of infection (p<0.001), also there was a positive relationship between having family members who had been infected with COVID-19 with vaccination acceptance rate (p=0.001), and respondents who had children (<18 years) seemed to show a statistically higher level of vaccination acceptance (p=0.035) ( Table 2).

**Table 2.** Medical history and its association with COVID-19 vaccine acceptance rate.

Parameters		n (%)	COVID-19 vaccine acceptance		p-value
			Yes	No	
Underlying medical condition	Positive	423 (34.6)	263	160	0.002
	Negative	798 (65.4)	423	375	
Personal history of COVID-19 infection	Positive	574 (47.0)	375	199	0.000
	Negative	647 (53.0)	311	336	
Family members infected with COVID-19	Positive	815 (66.7)	484	331	0.001
	Negative	406 (33.3)	202	204	
Underaged children	Yes	880 (72.1)	509	371	0.035
	No	341 (27.9)	177	164	

COVID-19, coronavirus disease 2019.

**Figure 1.**
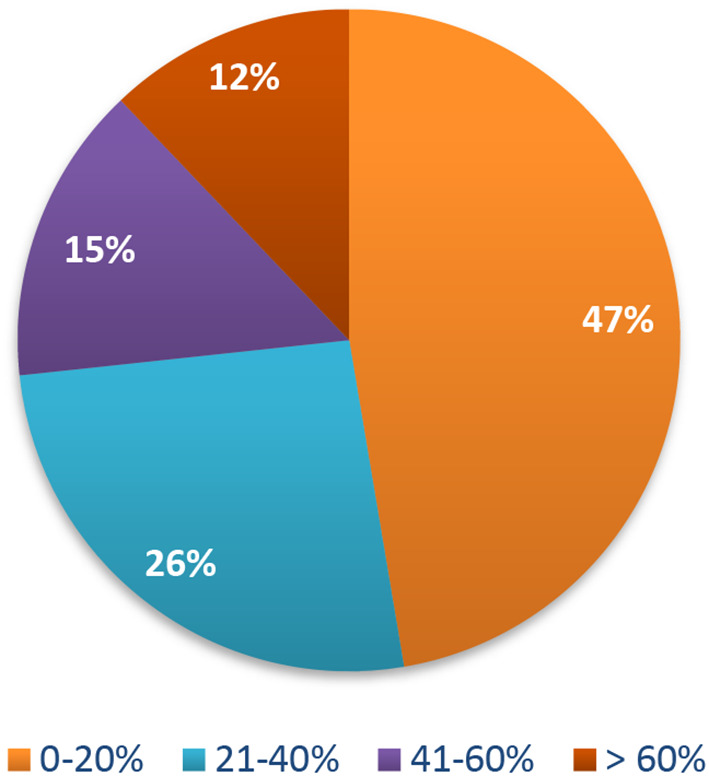
Risk perception of getting COVID-19. COVID-19, coronavirus disease 2019.

The percentage of perceived risk of getting COVID-19 was very low; the majority of participants (47%) believed their risk ranged from 0 to 20% ( Figure 1).

Table 3 depicts the study population’s understanding of COVID-19 vaccinations; most respondents thought that the priority of vaccination should be focused on health care providers.

**Table 3.** Public's understanding of COVID-19 vaccinations.

Factors	Answer	n (%)
How essential do you think the COVID-19 vaccination is?	Important	1040 (85.2)
	Not important	181 (14.8)
COVID-19 vaccine must always be mandatory for everyone	Agree	775 (63.5)
	Disagree	446 (36.5)
COVID-19 vaccination should always be mandatory for healthcare providers	Agree	935 (76.6)
	Disagree	286 (23.4)
The vaccine's approval ensures its safety	Agree	930 (76.2)
	Disagree	291 (23.8)
Receiving the immunization is the most effective COVID-19 prevention strategy	Agree	896 (73.4)
	Disagree	325 (26.6)

COVID-19, coronavirus disease 2019.

**Figure 2.**
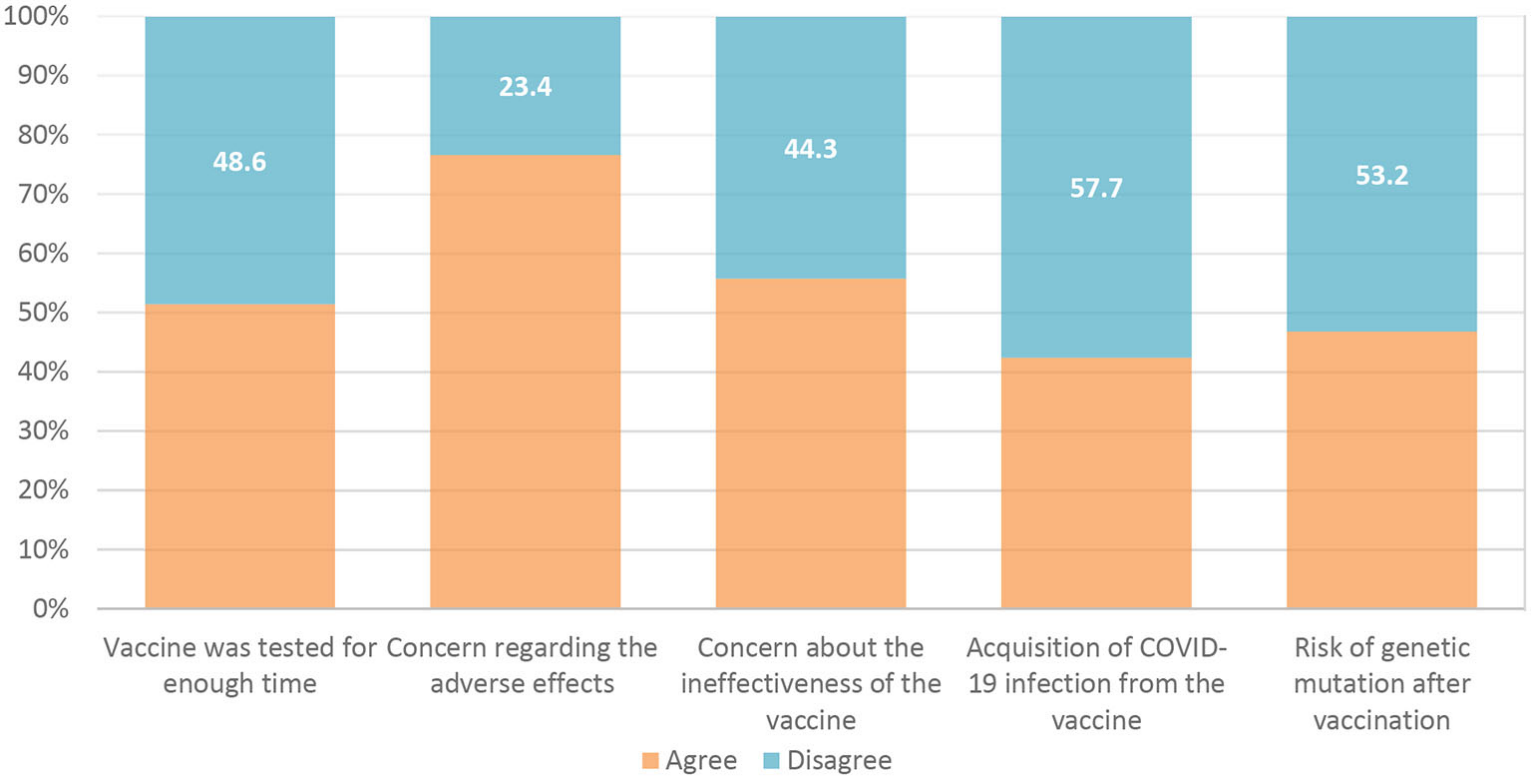
Barriers to receiving COVID-19 vaccine. COVID-19, coronavirus disease 2019.

The barriers to receiving the COVID-19 vaccine were exemplified by the vaccine not being evaluated for a sufficient period in 51.4% of the responses, as well as concerns about future side effects in 76.6% of the responses, and a lack of efficacy in 55.7% of the respondents ( Figure 2).

**Figure 3.**
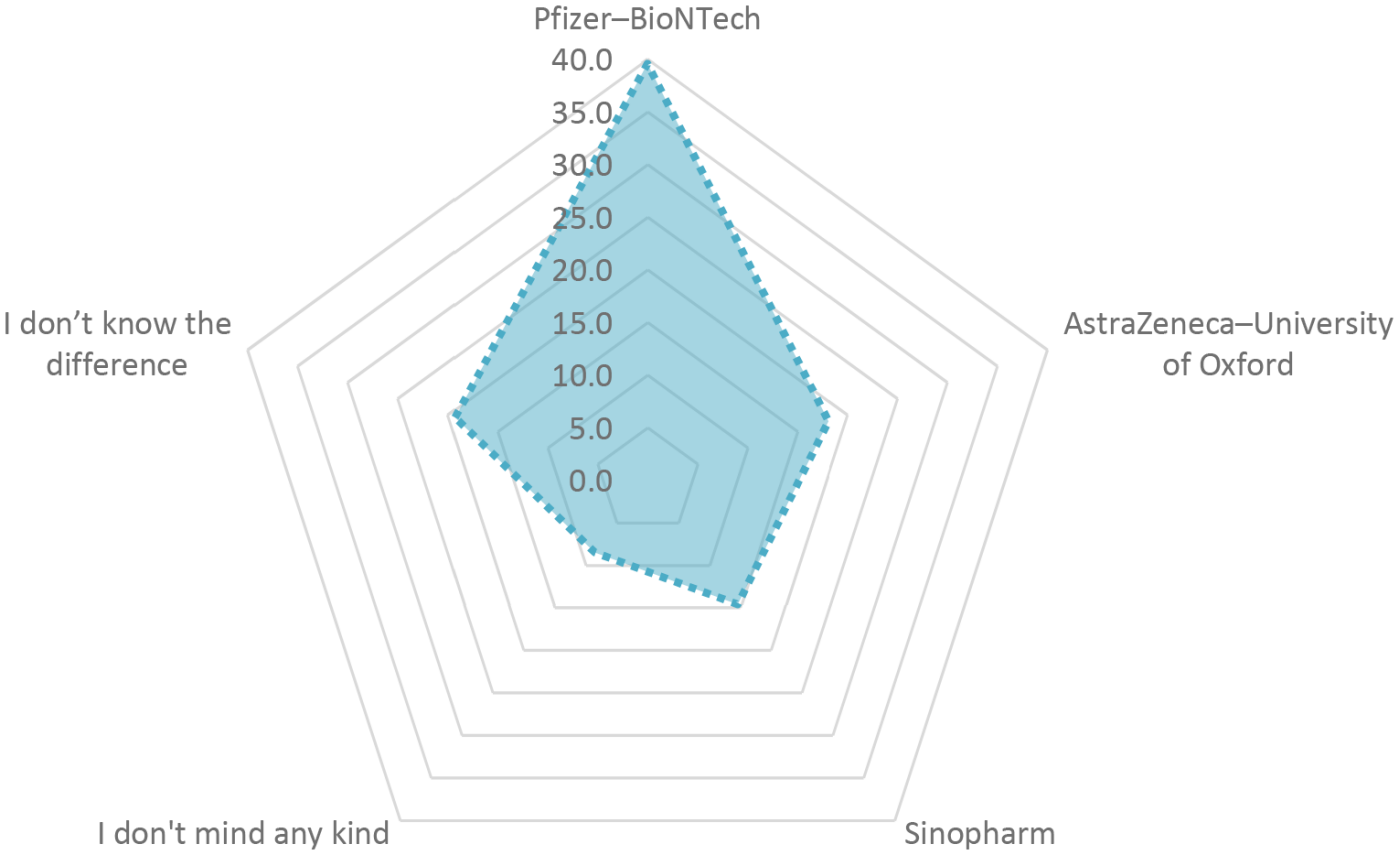
Vaccine preference.

The Pfizer-BioNTech vaccine received 39.6% preference and participants confidence followed by Oxford/AstraZeneca vaccine at 18.1% and the Sinopharm vaccine at 14.6%, as shown in Figure 3.

**Figure 4.**
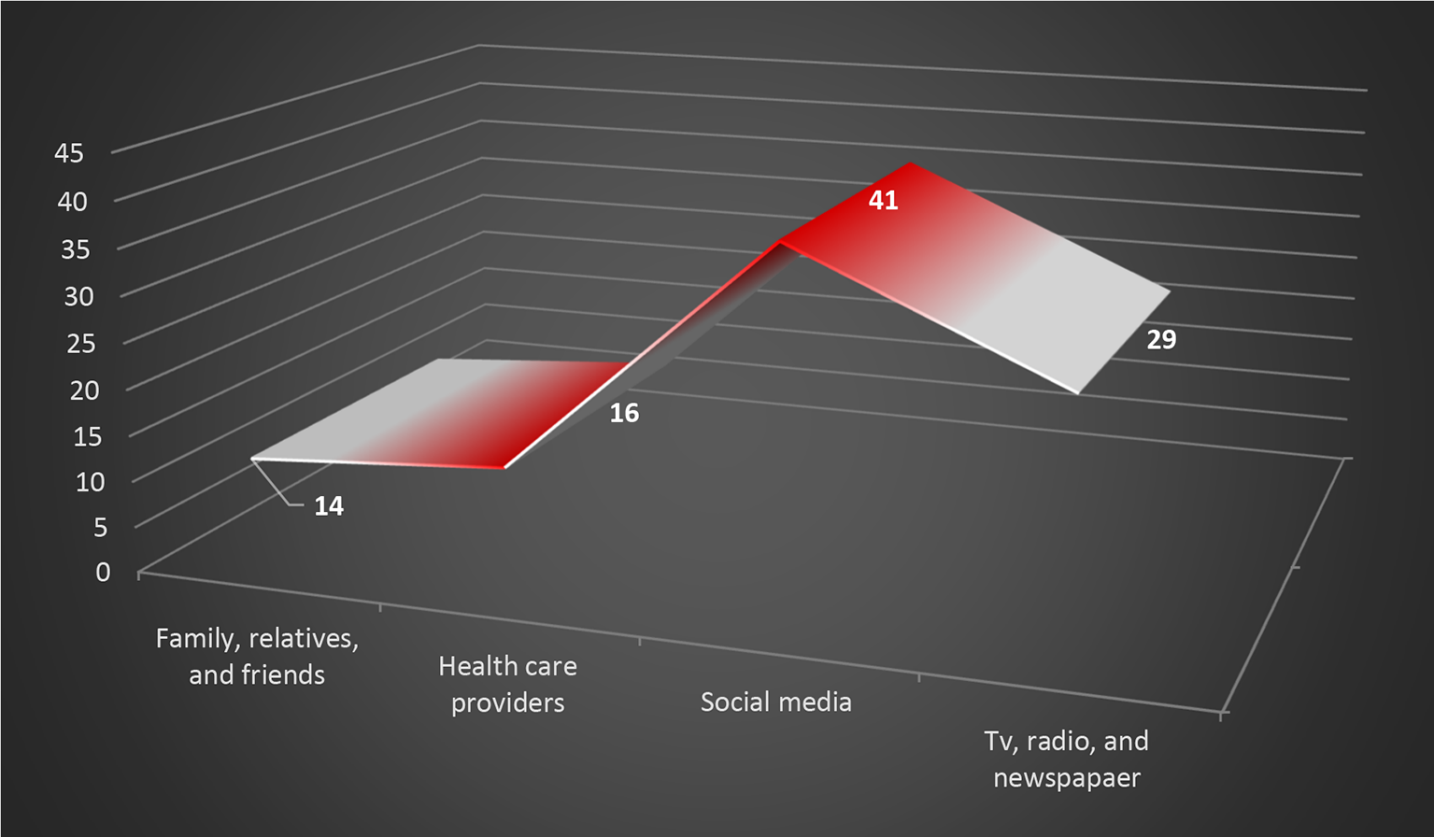
Graphical illustration for the primary source of COVID-19 information. COVID-19, coronavirus disease 2019.

Besides the governmental platforms, social media have become a significant source for COVID-19 information; according to the findings of this study, 41% of respondents obtained medical instruction from social media, as illustrated in Figure 4.

## Discussion

The vaccine is recognized as an important public health innovation of the 21st century. Its acceptability, however, varies with geography, time, socioeconomic class, ethnicity, and human-environmental behavior. ^27^ Despite the fact that there have been few studies to investigate the intention to use the COVID-19 vaccination during the current crisis in Iraq, our findings contradict those of studies conducted in China and the United States. ^28^^,^^29^ According to a study in China, 72.5% of members of the public intended to get the COVID-19 vaccine. ^29^ In addition, studies conducted in the United States indicated that the COVID-19 vaccine was accepted by 80% of the eligible population. ^28^ Throughout our research, 56.2% of participants indicated a desire to accept the COVID-19 vaccination. This study discovered there was a significant disparity in vaccination hesitancy frequency between men and women; however, vaccine hesitancy was high in both sexes.

Furthermore, the individuals who were reluctant to get the COVID-19 vaccine were older. According to Robertson *et al*., in the United Kingdom, 21% of women and 14% of men were apprehensive about getting the COVID-19 vaccine. ^30^ In contrast to this research, the younger respondents were less likely to refuse the COVID-19 vaccine.

Vaccine reluctance could be caused by a variety of scenarios, including disinformation and conspiracy beliefs. ^31^^,^^32^ Furthermore, health inequities, economic constraints, racial profiling, and access hurdles are seen as underlying reasons for poor trust and poor uptake. ^33^ According to Paul *et al*., in the United Kingdom, 16% of the study participants showed high levels of skepticism about the COVID-19 vaccines. ^34^ Minority groups, individuals with lower levels of education, lower yearly income, and a lack of knowledge of COVID-19, and those who do not follow government COVID-19 recommendations have higher levels of distrust. They reported that 14% of respondents had chronic diseases and 23% of this chronically ill population were unsure about receiving the COVID-19 vaccine. Our participants in this study had a greater prevalence of chronic illnesses (34%) and a higher rate of vaccination unwillingness. Meier *et al*., indicated that people who perceived themselves to be more susceptible to COVID-19 were more likely to have a COVID-19 vaccine in the United States, which is consistent with the results in our research. ^35^ The majority of study participants said that COVID-19 vaccination was necessary, especially for health care workers, and that it should be made mandatory once it was widely available. Corresponding findings were published by Lucia *et al*. ^36^ The Centers for Disease Control and Prevention (CDC) advises that medical practitioners receive the first doses of COVID-19 vaccines because of the higher risk of exposure. ^37^

The public’s confidence in the COVID-19 vaccine may fluctuate depending on the vaccine type. Research, for example, discovered the greatest degree of confidence in mRNA technology. Compared to AZD1222, both BNT162b and mRNA1273 had a better level of acceptability. ^38^ Furthermore, cases of bleeding, thrombosis and platelets dysfunction following COVID-19 vaccinations in individuals with preexisting hemodynamic abnormalities or those taking particular drugs have sparked widespread alarm on social media. As a result, the use of the Oxford/AstraZeneca vaccine has been temporarily suspended in various European nations. ^39^ Some officials were hesitant to receive the Oxford/AstraZeneca vaccine (ChAdOx1 nCov19) in May 2021 in Iraq. These officials awaited and received the Pfizer-BioNTech vaccine, which is based on mRNA. This unresponsive conduct may have contributed to apprehension about receiving the COVID-19 vaccine in Iraq.

The evidence shows that it is crucial to concentrate on building confidence in COVID-19 vaccines. This includes negotiating the COVID-19 evidence framework with credible communicators and fostering vaccination confidence via transparency and anticipatory monitoring. Societies must be included early on to respond to complaints, respond to questions, and dispel misconceptions. ^40^ Because public trust in vaccination is low, COVID-19 immunization programs can only succeed if there is a widespread conviction that the vaccinations offered are safe and effective. ^41^ Lucia *et al*., emphasized the importance of transparency to address concerns regarding the efficiency and safety of vaccine development. ^36^ It is critical to support COVID-19 immunization through public statements and news releases, as well as to monitor and combat false news. ^36^

## Conclusions

COVID-19 vaccination apprehension was discovered in almost half of the study population. Lack of understanding about vaccination eligibility, anxiety about adverse events and vaccine efficacy, and mistrust in the government were all independent predictors of vaccination hesitancy. A heightened risk perception of COVID-19 reduced vaccine hesitancy. Concerns were raised about a lack of vaccination-related information and the vaccine launch before the release of evidence on safety and effectiveness. While vaccination indecision has decreased over time, health education programs designed to raise vaccine awareness and build trust in government authorities would be beneficial.

### Limitations

Our study was limited by the fact that it was conducted after COVID-19 vaccination had been initiated. As a consequence, it is probable that it underestimated the initial vaccine rejection among individuals who were vaccinated and then moved to the vaccination accepting group.

## Data availability

### Underlying data

Zenodo: ‘Questionnaire responses for 1221 participants’. https://doi.org/10.5281/zenodo.6345333. ^42^

This project contains the following underlying data:

data zenodo.xls (Questionnaire responses for 1221 participants)

Data are available under the terms of the Creative Commons Attribution 4.0 International license (CC-BY 4.0).

### Extended data

Zenodo: ‘Research questionnaire’. https://doi.org/10.5281/zenodo.6345361. ^43^

This project contains the following extended data:

- Questionnaire.pdf

Data are available under the terms of the Creative Commons Attribution 4.0 International license (CC-BY 4.0).
