# Supplementary material for: COVID-19 vaccination hesitancy among Iraqi general population between beliefs and barriers: An observational study
Source: F1000Res. 2022 Apr 25;11:334. Originally published 2022 Mar 18. [Version 2] doi: 10.12688/f1000research.110545.2 (PMC9046899; doi:10.12688/f1000research.110545.2)
Supplement: Peer review history

# Peer review: Reviewer response for version 2

**DOI:** 10.5256/f1000research.133399.r135812

**Published:** 2022-04-27

**Authors:** Manal Mohammed Younus

**Affiliations:** 1 Ministry of Health, Iraqi Pharmacovigilance Center, Baghdad, Iraq

Thank you for re-sharing the manuscript after taking into consideration my previous notes. I went through the revised manuscript and the authors have now addressed all the concerns previously raised. I have no further concerns.

Is the work clearly and accurately presented and does it cite the current literature?

Yes

If applicable, is the statistical analysis and its interpretation appropriate?

Yes

Are all the source data underlying the results available to ensure full reproducibility?

Yes

Is the study design appropriate and is the work technically sound?

Yes

Are the conclusions drawn adequately supported by the results?

Yes

Are sufficient details of methods and analysis provided to allow replication by others?

Yes

Reviewer Expertise:

Pharmacovigilance and pharmacoepidemiology, drug safety, clinical pharmacy and pharmacy practice.

I confirm that I have read this submission and believe that I have an appropriate level of expertise to confirm that it is of an acceptable scientific standard.

# Peer review: Reviewer response for version 1

**DOI:** 10.5256/f1000research.122167.r128019

**Published:** 2022-04-19

**Authors:** Manal Mohammed Younus

**Affiliations:** 1 Ministry of Health, Iraqi Pharmacovigilance Center, Baghdad, Iraq

First, I would like to thank you for the opportunity to review this article. The subject is important, and there is still a need to have such studies covering it.

 The manuscript describes the responses of online respondents to the survey items tackling different factors that reflect the understanding of the respondents to the COVID-19 vaccination and barriers to receiving the vaccines. The study also included the COVID-19 vaccines preferences and the primary source of information related to the vaccines. The study showed that about half of the participants had hesitancy in accepting the vaccines. The barriers were related to the vaccine's eligibility, effectiveness, and safety.

 Below are my notes:

1. Introduction: 

- Please rewrite the section with references (4-13) and tell in a more clear way how the problem is not only H1NI vaccine-related. 
- There is no mention of COVID-19 vaccines in this paragraph, and one would assume that all that is written is related to H1N1 vaccine hesitancy. Please clarify.

2- Methods:

- Regarding the questionnaire, please mention if internal or external validation was required and if it was adopted from other languages.
- Also, why do you use only two options yes or no, and agree or disagree, and not give a third alternative option like I don't know?
- Regarding the statistical analysis; could you elaborate on why using Fisher’s exact test when your sample size is big enough to be analyzed using chi-square.
- In the last two lines of the data analysis part; the manuscript said "If data are missing for over 60% of the samples, it may be best to remove if the variable is insignificant." Is this a statement or it is the methodology that was used by the researcher. Please clarify.

3- Results;

- Could you please mention what social media platform you distributed the questionnaire to and how many responses you received throughout the email! 

4- Discussion; 

- Please insert a reference to the paragraph; "Some officials were hesitant to receive the Oxford/AstraZeneca vaccine (ChAdOx1 nCov19) in May 2021 in Iraq. These officials awaited and received the Pfizer-BioNTech vaccine, which is based on mRNA. This unresponsive conduct may have contributed to apprehension about receiving the COVID-19 vaccine in Iraq."

Is the work clearly and accurately presented and does it cite the current literature?

Yes

If applicable, is the statistical analysis and its interpretation appropriate?

Yes

Are all the source data underlying the results available to ensure full reproducibility?

Yes

Is the study design appropriate and is the work technically sound?

Yes

Are the conclusions drawn adequately supported by the results?

Yes

Are sufficient details of methods and analysis provided to allow replication by others?

Yes

Reviewer Expertise:

Pharmacovigilance and pharmacoepidemiology, drug safety, clinical pharmacy and pharmacy practice.

I confirm that I have read this submission and believe that I have an appropriate level of expertise to confirm that it is of an acceptable scientific standard, however I have significant reservations, as outlined above.

# Response

**Published:** 2022-04-20

**Authors:** Laith G. Shareef

**Affiliations:** AL-ESRAA University College, Iraq

I appreciate the time and effort the reviewers have dedicated to providing valuable input on my manuscript. I have been able to incorporate changes to reflect most of the suggestions provided by the reviewer, and I have highlighted the changes within the manuscript.

 Introduction:

- I’m sorry for being misunderstood, I rewrote the paragraph with references (4-13) as you recommended, and please let me clarify my point of view for this section to be clearer for you and the readers: 
  This section summarized the acceptance rate of previously developed vaccines not only in H1N1 in USA and UK (Ref. 5 & 6); but also, in AH7N9 in China (Ref. 8), seasonal flu immunization in Ireland among medical staff (Ref. 10 & 11), refusal of pediatric age group vaccination in the Arab region (UAE) (Ref. 12), and the association of flu vaccine acceptance with the COVID-19 vaccine acceptance (Ref. 13).
- Thank you for your recommendation regarding adding more about COVID-19 acceptance, I reinserted more about this topic in reference 17.  

Methods:

- Thank you for your comments regarding validations and other languages usage, the required details have been mentioned in the revised version.
- Thank you for your comments regarding the type of question. The questions in this study were adopted from the book "Questionnaire Design: Asking Questions with a Purpose," https://vulms.vu.edu.pk/courses/HRM619/Downloads/How_to_ask_questions_through_questionaire.pdf and we discovered that one of the permissible kinds of close-ended questions is as follows:  "there is a variety of ways to write close-ended questions. Some required answers that fall along an implied continuum (as in a rating scale); others supply answers in no particular order (lists): ( check all that apply); others provide relevant answers but allow respondents to add others not on the list. the following section gives examples of two-options responses: No-Yes, Disagree-Agree, False-True, and Oppose-Favor."
- Thank you for your comments regarding the tests used for data analysis. I reviewed more than statistical references comparing the two tests listed above: -
  1) As the name indicates, Fisher's exact test provides an exact right result regardless of sample size. https://www.graphpad.com/guides/prism/latest/statistics/stat_chi-square_or_fishers_test.htm
  2) When the sample size is high enough, the chi-square p-value approaches that of a Fisher's exact. Fisher's exact also has the advantage of being applicable to large sample sizes. https://www.saem.org/about-saem/academies-interest-groups-affiliates2/cdem/for-students/cdem-voice/educational-research-column/educational-research-column-choosing-wisely-chi-square-vs.-fisher-s-exact
- Thank you for your comments regarding the manuscript's last two lines in the data analysis section. Actually, this was how to handle missing data.

Results:

- Thank you for your comments regarding questionnaire distribution.
  As you recommended, we clarified the revised version of the manuscript, the questionnaire has been distributed throughout the most widely used social media in Iraq, like Facebook, Twitter, and Instagram. We did not get the responses by email we only distribute the questionnaire through it to relatives or co-workers who did not have been in touch over the social media.

Discussion

- Thank you for your comment regarding inserting the reference for the aforementioned text. This was a piece of trending news over the newspapers, tv news since no reliable reference could be cited, I removed the paragraph from the revised version.

# Peer review: Reviewer response for version 1

**DOI:** 10.5256/f1000research.122167.r128221

**Published:** 2022-04-01

**Authors:** Reem Hamdy A. Mohammed

**Affiliations:** 1 Rheumatology Department, Faculty of Medicine, Cairo University, Cairo, Egypt

The given study is a descriptive observational research study that aimed to investigate the public perception of COVID-19 vaccination in the Iraqi population and it's relation to differing participation, as well as vaccine related variables that might affect perception, acceptance and decision to vaccinate.

 The authors used a google form constructed survey divided into three sections, each concerned with specific variables that relate to the domains of concern. In building their research questionnaire and defining the sample size authors used data from the literature discussing the question of research going through detailed revision of similarly published international data as an evidence based reference. The analysis done was informative, focusing on the variables to be addressed and providing thorough descriptions of the relationships intended to be explored, including comments on the perception and acceptance for the different forms of vaccinations available.

 Authors concluded from their questionnaire that lack of sufficient data about the potential long-term benefit versus risk of the vaccination constitutes the cornerstone behind hesitancy to vaccinate. The study is interesting and adds to the body of knowledge regarding the impact of the pandemic and the preventive strategies designed.

Is the work clearly and accurately presented and does it cite the current literature?

Yes

If applicable, is the statistical analysis and its interpretation appropriate?

Yes

Are all the source data underlying the results available to ensure full reproducibility?

Yes

Is the study design appropriate and is the work technically sound?

Yes

Are the conclusions drawn adequately supported by the results?

Yes

Are sufficient details of methods and analysis provided to allow replication by others?

Yes

Reviewer Expertise:

Autoimmune diseases, clinical immunology.

I confirm that I have read this submission and believe that I have an appropriate level of expertise to confirm that it is of an acceptable scientific standard.
